# Opioids and Chronic Pain: An Analytic Review of the Clinical Evidence

**DOI:** 10.3389/fpain.2021.721357

**Published:** 2021-08-17

**Authors:** Stephen E. Nadeau, Jeffrey K. Wu, Richard A. Lawhern

**Affiliations:** ^1^Research Service and the Brain Rehabilitation Research Center, Malcom Randall VA Medical Center and the Department of Neurology, University of Florida College of Medicine, Gainesville, FL, United States; ^2^Cornell University, Ithaca, NY, United States; ^3^Independent Researcher and Patient Advocate, Fort Mill, SC, United States

**Keywords:** opioids, opioid efficacy, opioid dosage, opioid mortality, opioid use disorder, opioid crisis, opioid crisis causes

## Abstract

We conducted an analytic review of the clinical scientific literature bearing on the use of opioids for treatment of chronic non-cancer pain in the United States. There is substantial, albeit not definitive, scientific evidence of the effectiveness of opioids in treating pain and of high variability in opioid dose requirements and side effects. The estimated risk of death from opioid treatment involving doses above 100 MMED is ~0.25%/year. Multiple large studies refute the concept that short-term use of opioids to treat acute pain predisposes to development of opioid use disorder. The prevalence of opioid use disorder associated with prescription opioids is likely <3%. Morbidity, mortality, and financial costs of inadequate treatment of the 18 million Americans with moderate to severe chronic pain are high. Because of the absence of comparative effectiveness studies, there are no scientific grounds for considering alternative non-pharmacologic treatments as an adequate substitute for opioid therapy but these treatments might serve to augment opioid therapy, thereby reducing dosage. There are reasons to question the ostensible risks of co-prescription of opioids and benzodiazepines. As the causes of the opioid crisis have come into focus, it has become clear that the crisis resides predominantly in the streets and that efforts to curtail it by constraining opioid treatment in the clinic are unlikely to succeed.

## Introduction

The opioid crisis, already of staggering proportions, continues to grow despite many years of effort within the field of medicine, the issuance of treatment guidelines, and substantial legislative action across the nation. At the same time, we find ourselves at an impasse. On the one hand, we have the scientific knowledge to substantially address the crisis. On the other hand, the combination of efforts by physicians concerned with rising opioid mortality, the issuance of a national guideline by the Centers for Disease Control and Prevention (CDC) (1), and legislative action has not had a measurable impact on the crisis. Worse, it has spawned a second crisis (2), this one involving Americans who have relied for years on opioid treatment to manage chronic pain and enable them to contribute to society and enjoy some quality of life. Epidemiologic studies suggest that 22% of U.S. adults (55 million) experience chronic pain and 7% (18 million) moderate to severe pain (3). These patients now face disability, inordinate suffering, and excess mortality. Given these two crises, it seems timely to re-assess the scientific evidence and examine its implications for medical practice, public policy, and further research.

Our particular focus will be on issues relevant to clinical decision making by the practitioner; clarification of the research questions that need to be addressed; and clinical trial experimental designs that may be able to address questions in this field that have stymied conventional designs. Our review involved particularly careful analysis of study methodology and data with an attempt to incorporate the full dimensionality of chronic pain and its treatment in each assessment. Some perspectives on the opioid crisis have been substantially influenced by misperceptions [reviewed by Oliver and Carlson (4)].

This analysis is based almost entirely on American literature. There may be much for other countries to learn from the American experience. However, the particular characteristics of the opioid crisis in America reflect cultural influences, the extraordinary heterogeneity of American society, the existence of large pockets of poverty, the absence of comprehensive health care for every citizen, an American approach to opioid abuse that has emphasized interdiction and incarceration over mental health treatment, the availability of licit and illicit opioids, laissez faire approaches to business regulation (hence pill mills), and long-standing ambivalence among physicians to treatment of pain. They also reflect the prevalence of the particular hopelessness that comes from denial of opportunity to people living in a country founded on hope.

All clinical studies of opioids inevitably reflect the fact that opioid treatment may not be sustained and that it may be discontinued for a variety of reasons, including lack of efficacy, adverse effects, comorbidities, drug abuse, and lack of access to alternative treatments. From an analytic point of view, these factors contribute to unexplained statistical variance.

Meta-analyses have become the generally accepted means for evaluating the large clinical trial literature, even as such analyses often do not adequately consider the scientific strengths and weaknesses of individual trials, instead focusing almost entirely on the quantitative outcomes and their susceptibility to meta-analysis. Most critically, intention to treat designs (the gold standard for RCTs) involving patients with more severe pain are either seriously undermined or precluded by high drop-out rates in placebo groups. Avoidance of these high drop-out rates requires inclusion of only patients with modest pain, who are less likely to benefit, while accommodating the limited dose titration that is possible in short duration trials (5). The particular focus on patients with modest pain is reflected in the modest doses of opioids typically employed. Of the 96 trials reviewed by Busse et al. (5), 35% involved tramadol and in the 87 RCTs for which dosing data were quantified, median milligrams morphine equivalent/day (MMED) was 45 (interquartile interval 28.2–78.3).

## The Efficacy of Opioids in Treatment of Chronic Pain

A large number of randomized placebo-controlled trials (RCTs) have been conducted to test the efficacy of opioids in treatment of chronic non-cancer pain (5–8). Taken together, they provide evidence of modest opioid efficacy in relief of pain and improvement of physical functioning but also significant opioid side effects. Unfortunately, by and large, these trials have been marked by failure to accommodate the enormous patient to patient variability in necessary opioid dosage (see below), failure to titrate opioids to achieve adequate control of pain, over-rapid drug titration (which magnifies side effects and renders achievement and assessment of dosage adequacy difficult), and lack of recognition of the high prevalence of idiosyncratic side effects (9, 10). It may take many months to identify an opioid that is well-tolerated by a given patient, gradually titrate dosage to the point of effective control of pain, and effectively treat important comorbidities such as depression. However, among the 62 trials reviewed by Furlan et al. (6), 51% were one month or less, 39% were 5–12 weeks in duration, and the remaining 9% were 13–24 weeks in duration. There are several reports of open trials, non-randomized, involving large numbers of patients treated with either transdermal fentanyl or oxycodone continuous release that have demonstrated the ability to achieve sustained relief of pain for years (11–14). Although these trials provide some evidence of long-term efficacy and low incidence of tolerance, they cannot substitute for RCTs. In sum, few trials employing rigorous scientific methods have tested opioids as they are best used in clinical practice (15).

The challenges of testing opioid effectiveness in a way that can translate readily to clinical use can be addressed by employing an Enriched Enrollment Randomized Withdrawal (EERW) design. A 3-month trial of extended release oxymorphone for chronic moderate to severe low back pain, conducted by Hale et al. (16), involving 250 patients, is representative. During the first phase of the trial, oxymorphone was titrated to clinically optimal dosage and participants intolerant of the drug dropped out. Those stabilized on oxymorphone (*N* = 143) were then randomized to drug continuation or placebo. Physical withdrawal symptoms in those randomized to placebo were mitigated with supplementary oxycodone. By 3 months, 75% of patients in the placebo group had dropped out (53% from lack of efficacy; 11% from side effects; 11% other), compared with 30% of the oxymorphone group (11% for lack of efficacy; 10% from side effects; 9% other), thereby providing substantial evidence of efficacy. However, the high placebo drop-out rate obviated intention to treat statistical analysis of pain scores. At the end of the titration phase, 72% of patients rated their experience with the oxymorphone as good or excellent. Other EERW trials have achieved comparable results (17–20); see also review (21) and meta-analysis (22). This said, EERW trial results, in aggregate, suggest the possibilities rather than proving the case.

In addition to addressing the challenges of emulating opioid prescription in good clinical practice, EERW trials have analytic advantages and achieve greater statistical power (23). Visual analog pain scales (VAPS), the typical primary outcome measure in opioid RCTs, may be, like subjective measures in general, susceptible to anchor point drift over time (24). They also correlate poorly with more objective measures of pain, such as the McGill Pain Questionnaire (25). With an EERW design, efficacy can be established with a logistic outcome measure—participant drop-out, thereby turning to advantage the dropout problem that plagues trials of conventional design. Drop-out may occur because of inadequate control of pain or because of opioid side effects.

Scant data are available on the distribution of opioid dosage typically needed to achieve adequate control of pain. In an EERW trial of oxymorphone for treatment of chronic low back pain involving 325 participants, Katz et al. (18) reported that 76.8% of those who successfully completed the oxymorphone titration phase (*N* = 205) achieved ≥30% pain reduction and 67.4% experienced a >50% decrease in pain; 97% rated the treatment as good, very good, or excellent. Among participants, 53% had been titrated to ≤90 mg morphine equivalent/day (MMED), 81% to ≤150 MMED, and 93% to ≤240 MMED. Maximum dose in the trial was 420 MMED [see also Rauck et al. (19)].

The RCT conducted by Krebs et al. (26), which involved 240 patients treated for chronic pain in VA hospitals, has been widely cited as proof that opioids are no more effective than non-opioid pharmacologic treatments for chronic pain. However, the mean dose of opioid was 21 MMED and only 12.6% of patients randomized to the opioid group were taking >50 MMED. Furthermore, antidepressants were among the treatment options in the non-opioid group. These study details suggest that the results of this trial may be best construed as: (1) patients whose pain is not sufficiently severe to warrant opioid treatment do not particularly benefit from opioids; or (2) opioids are not of benefit to patients with moderate to severe chronic pain when opioid dosage is not sufficiently titrated; or (3) the optional use of antidepressants in the non-opioid group substantially mitigated the inadequacy of other non-opioid therapy.

Given that further clinical trials are needed, we propose a variation on the EERW design in which initial dose is very gradually titrated and participants, rather than being randomized to drug continuation or placebo, are randomized to continuation of their opioid regimen without change or to gradual tapering, e.g., by 10%/month, utilizing control tablets containing less and less opioid—an enriched enrollment, randomized *gradual* withdrawal design (EERGW). The statistical method would be survival analysis based upon time to trial drop-out (27). This design would likely be more successful than EERW designs in sustaining participant blinding. It would enable trials extended over almost arbitrarily long periods of time and the use of Cox proportional hazards analysis to identify potential predictors of outcomes.

## One Dose Fits All

The concept that one dose fits all has arguably been the single recommendation of the CDC that has had the greatest negative impact on patients in chronic pain, even as a number of studies suggest that the concept is not valid.

Data from EERW trials suggest 13-fold dosage variability (16, 18). These results are congruent with those of multiple studies of management of post-surgical pain in opioid-naïve patients, which have revealed an ~15-fold variability in opioid dose requirements (28–31). This experience with opioid-naïve patients suggests that dose-variability is a phenotypic phenomenon and not simply related to tolerance.

The reasons for the high variability in opioid dosage needed to achieve control of chronic pain are not well-understood. Severity of pain must be a factor. Genetic differences in hepatic metabolism can account for 3-fold or greater variability (32, 33). Genetic differences in the receptor interactions of different opioids (34) and in neural transmission also appear to be important (35, 36).

## Risk of Death From Opioid Treatment

**Figure 1:**
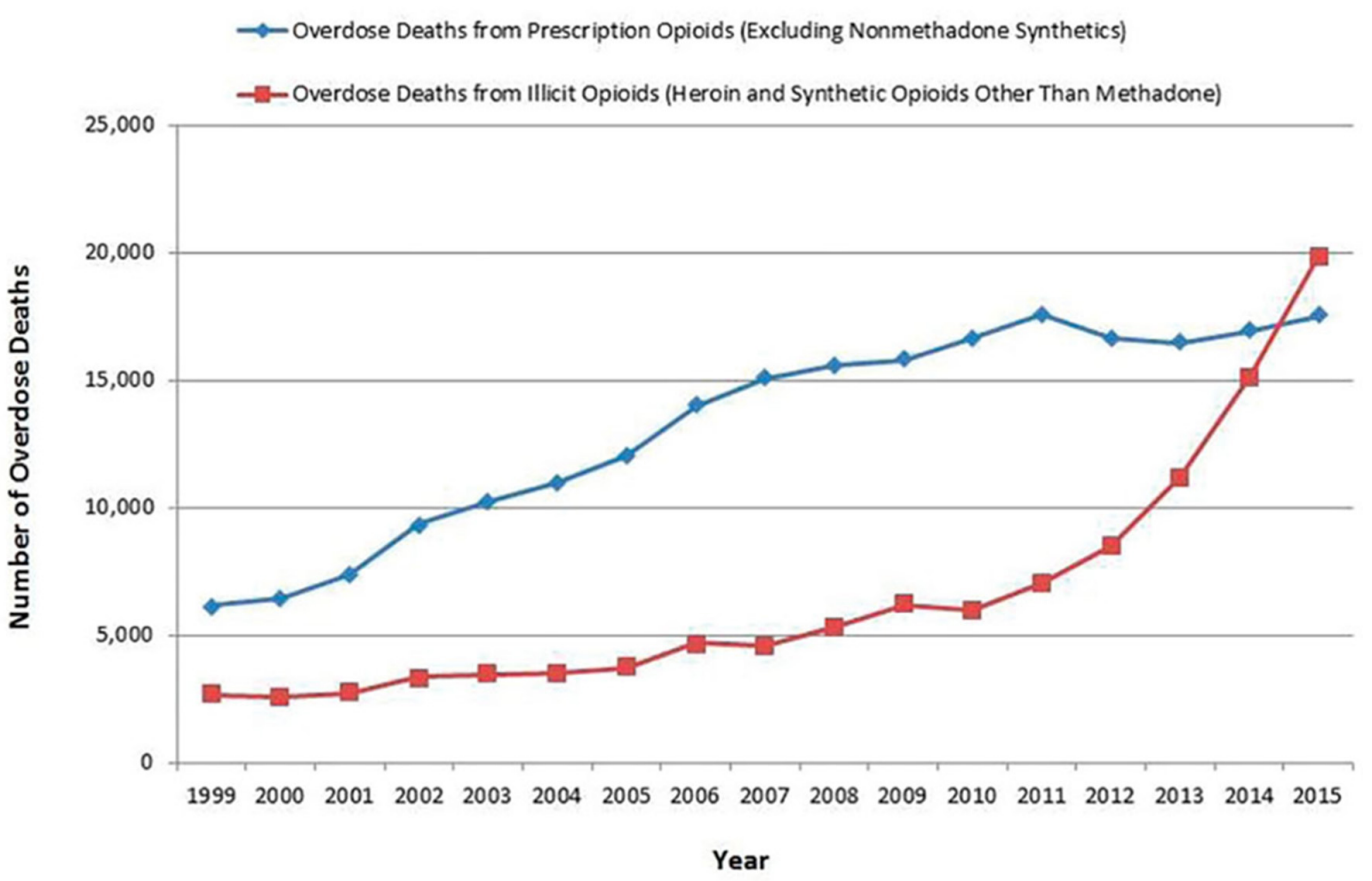
Number of overdose deaths from prescription and illicit opioids, United States, 1999–2015 (37).

The rise in prescription opioid-associated mortality from ~6,500/year in 1999 to 17,500/year in 2011 (37) (Figure 1) generated widespread concern about the risks of opioid use and paved the way for the idea that opioid over-prescribing was responsible for the opioid crisis. However, two things have been missing from this conversation: (1) the statistical contribution of increasing numbers of patients being prescribed opioids; and (2) the number of annual deaths related to prescribing by pill mills, in which opioid use is not adequately medically supervised.

It is absolute risk, not proportional risk, that matters for an individual patient and practitioner when considering a treatment (38). The estimated annual opioid-associated case fatality rate with prescription of >100 MMED is 0.25% (39) and rises to 0.5% in those receiving >400 MMED (40).

Results of epidemiologic studies are congruent with these findings. In the North Carolina study of Dasgupta et al. (41), the estimated annual mortality associated with oxymorphone (the drug with the highest associated mortality) was 0.54%/year. In this study, annual mortality rose more or less linearly with opioid dose (without an inflection point), reaching a maximum of 0.80% (95% CI 0.55–1.10) at 650 MMED. This study could not distinguish between deaths associated with opioids prescribed chronically for treatment of chronic pain and deaths associated with “one-off” prescriptions obtained by opioid abusers on the other. Only 51% of decedents had an active opioid prescription on the day of death and 24% had no record of being dispensed an opioid in the prior year, a finding replicated in other studies (42, 43).

It may be challenging to distinguish mortality related to opioids *per se* from mortality associated with opioids and conditions under which they are prescribed (in which case, to one extent or another, opioid prescription may be simply a marker of disease and condition) (see also below: Morbidity and mortality associated with chronic pain). Agnoli et al. (44) assessed *all-cause* mortality among 90,622 participants in the Medical Expenditure Panel Survey according to whether patients had received no opioids, 1–5 opioid prescriptions, or six or more opioid prescriptions during the first year of 2-year study epochs. In the unadjusted analysis, there was a strong association between opioid prescriptions and mortality. However, this association disappeared when the analysis was adjusted for socio-demographics, health status, and health care utilization.

The prevalence of chronic pain, coupled with these case fatality rates, poses what may be a unique conundrum for medicine and public policy. If there were 10 million Americans with chronic pain who required opioid dosage of >100 MMED to achieve adequate pain control, this would translate to an annual mortality of 25,000. What may be acceptable to the patient and constitute responsible individualized treatment of a serious health problem by a physician thus may scale up to an issue that intrinsically warrants national concern.

Almost certainly, prescription opioid case fatality rates, however modest, could be further reduced by better training of physicians (45), more complete eradication of pill mills and black-market sources of opioids, reduced prescription-opioid diversion, better ascertainment and treatment of comorbid depression, reduction of all too frequent concurrent abuse of alcohol (46), and a better understanding of why overdoses occur (47).

The data reviewed here on opioid benefits, however incomplete, and risks provide the basis for opioid treatment decisions based upon a careful weighing of benefits against risks, as with medical decision making in general. In medical practice, we commonly weigh risks and benefits that are comparable to those associated with chronic opioid therapy. For example, the case-fatality rates associated with >100 MMED opioid therapy are comparable to the risks of fatal bleeding associated with use of rivaroxaban (0.2%/year) and warfarin (0.5%/year) in the prophylaxis of stroke due to atrial fibrillation (48). This might be considered an inapt comparison. However, systemic anticoagulation for atrial fibrillation is recommended for CHA_2_DS_2_-VASc scores of ≥2 (49), which corresponds to an annual stroke risk of ≥2.2% (50). The 5-year likelihood of being stroke free in a patient with the 2.2% annual stroke risk is 89.5%. On the other hand, the patient with moderate to severe chronic pain experiences suffering and disability from the outset.

## Prevalence of Opioid Use Disorder

Opioid use disorder (OUD) is thought to be prevalent among patients prescribed opioids (51). The extent to which clinicians make the diagnosis of OUD on the basis of perception of suspicious behavior, e.g., requests for increased opioid dosage to ease pain [“pseudo-addiction” (52, 53)], as opposed to DSM criteria, is unknown, and diagnoses based solely on clinician judgment must therefore be questioned. The prevalence of pseudo-addiction warrants further study. Vowles et al. (54), in an oft-cited study, reviewed a carefully selected 38 papers from a total of 367 identified in the literature. These papers reported rates of misuse of 0.08–81% (1,012-fold variability), abuse of 8% (data provided by one study) and ostensible addiction of 0.7–34.1% (48.7-fold variability). Incidence of iatrogenic opioid abuse (ICD-9 or DSM-4 criteria) is lower in studies of higher quality; studies using ICD-9 criteria compared with DSM-4 criteria; the use of strong opioids; and with prescriptions of ≥ 3 months duration (55). Other reviews, for example that of Fishbain et al. (53), which included 67 studies, revealed variability between studies comparable to that reported by Vowles et al. a mean rate of ostensible addiction of 3.27%, and a complicated and nuanced picture of opioid use and misuse in patients on opioid therapy for chronic non-cancer pain.

The enormous variability in results reported by Vowles et al. (54) raises questions about the validity and reliability of the outcome measures. The definitions of the outcome measures provide some clues to potential sources of the variability. Misuse was defined, according to widely accepted criteria, as opioid use contrary to the directed or prescribed pattern of use, regardless of the presence or absence of harm or adverse effects. This definition could be applied in several ways unrelated to abuse: patient use of the opioid at times of the day at odds with those recommended by the prescriber, taking extra pills of short acting drugs on bad days and less than the prescribed amount on good days (pain may fluctuate substantially from day to day), urine drug screens that were either falsely positive or turned up marijuana use, requests for an increase in opioid supply to cover inter-current surgery, accidents (however rare), or single instances of use of a different opioid diverted from a family member. We suggest that in good clinical practice, a judgment of misuse should hinge on patterns of behavior extending over repeated clinic visits. Addiction was defined by Vowles et al. (54) as “impaired control over drug use, compulsive use, continued use despite harm, and craving.” Addiction is an extraordinarily complex disorder and is operationally very difficult to define (56). A diagnosis of addiction could be correct. However, practicing clinicians are rarely in a position to apply DSM criteria for addiction in a fully informed manner. We suggest that in the present US regulatory environment, “potential for harm” may be as much in the eye of the prescriber or the pharmacist as in any observable behaviors of the patient. Physician concern is often dosage-related [e.g., >90 MMED since CDC 2016 (1)]. “Compulsive” use might simply reflect the severity of the pain and the inadequacy of pain control. “Craving” might actually reflect pseudo-addiction—the patient craves higher doses because pain control is inadequate.

A very different type of analysis of the prevalence of OUD by Han et al. (57), based on data from the 2015 National Survey on Drug Use and Health (NSDUH) is revealing. The sample consisted of 78,976 respondents aged 12 years or older living in households or non-institutional group housing who were representative of non-elderly US adults in 2015–2016. Data on sensitive questions were obtained through a computer driven audio interview arranged to assure anonymity. Weighted estimates suggested that 91.8 million (37.8%) of U.S. civilian non-institutionalized adults used prescription opioids over the prior year. Of these, 12.5% reported opioid misuse (use other than as directed by a physician) and 2.1% opioid abuse (defined as meeting ≥1 of four DSM-4 abuse criteria). Relief of pain was reported as the most common reason for opioid misuse (66.3%) and opioid abuse (48.7%). Among respondents who reported misuse or abuse, opioids were most often obtained from a physician (35.1 and 44.3%, respectively) or a friend or relative (53.1 and 35.9%) and were uncommonly obtained from a stranger or drug dealer (3.1 and 13.8%). The study by Han et al. suggests that opioid abuse is relatively rare among patients prescribed opioids (2.1%) and in 48.7% of cases, search for pain relief is the major driving factor.

Use of non-prescription opioids to medicate a health problem is strongly negatively correlated with ultimate heroin use (58). Boscarino et al. (59), in a large interview survey of a clinic population, reported a *lifetime* prevalence of mild OUD [2–3 DSM-5 symptoms (60)] of 28.1%, moderate OUD (4–5 symptoms) of 9.7%, and severe OUD (6+ symptoms) of 3.5%. It is worth noting that item 1 of the DSM-5 criteria (use of opioids in larger amounts or over a longer period than intended) would likely be endorsed by a large percentage of patients treated for chronic pain. For items 2 (persistent desire or unsuccessful efforts to cut down), 3 (a great deal of time spent in activities necessary to obtain the opioid), 4 (craving or strong desire to use opioids), and 7 (important social, occupational, or recreational activities are given up or reduced because of opioid use), responses could easily reflect a conflation of opioid effects with the effects of pain, desire to alleviate pain, activities involved in getting treatment for pain, or activities forgone because of persistent inadequately controlled pain. In a large survey of a clinic population, among participants who acknowledged only 2–3 symptoms (mild OUD), 33.7% endorsed item 1, 88.1% item 2, 44.7% item 4, and 23.8% item 7 (61). These data suggest a need to refine our operational measures of OUD [see Fishbain et al. (53) for extended consideration of this issue].

## The Gateway Theory

Up to 80% of patients reporting at least one past episode of heroin use also report at least one prior *nonmedical* use of prescription pain relievers (62). The word “non-medical” is often lost in discussion and the conclusion drawn that prescription of opioids, however brief, carries a high risk of leading to OUD and thus, constitutes a gateway to drug abuse. Acceptance of the gateway theory has also added fuel to the argument that many patients who are prescribed opioids are taking them because of OUD and not pain.

Several large studies refute the gateway theory. Brat et al. (63) reported a retrospective study based on insurance records of 1,015,116 opioid naïve patients undergoing surgery, 56% of whom received post-operative opioids. In the course of follow-up, 0.6% received a clinical diagnosis of opioid abuse during an average follow-up of 2.5 years. Likelihood of a diagnosis of opioid abuse was 0.15% among patients provided an opioid prescription for <1 week and rose to asymptotically approach 2% in patients prescribed opioids for >13 weeks. It is plausible that ongoing pain, rather than OUD, led to ongoing patient requests for opioid prescription renewals (pseudo-addiction), particularly given that the prevalence of persistent pain 6 months after surgery has been reported to be as high as 29.5% with some surgical procedures (64).

Sun et al. (65) reported a retrospective study of 641,941 opioid-naïve patients undergoing 11 common surgical procedures, including total knee arthroplasty (TKA), total hip arthroplasty, laparoscopic or open appendectomy, laparoscopic or open cholecystectomy, Cesarean section, sinus surgery, transurethral resection of the prostate, and simple mastectomy. The 1-year incidence of chronic opioid use (defined as 10 renewed prescriptions or 120 days of continuous use within 1 year) ranged from 0.09% for Cesarean section to 1.41% for TKA. The reported incidence of chronic opioid use in non-surgical patients was 0.136%. Shah et al. (66) reported a retrospective study of 675,527 patients who had undergone urologic surgery. Within the subsequent year, a documented clinical diagnosis of opioid dependence or overdose (i.e., without reference to DSM criteria) was made in 0.09%.

These studies, involving a total of 2,332,584 patients, suggest that the risk of long-term persistent use of opioids, or of clinically diagnosed abuse, following treatment for acute perioperative pain, is extremely low. They also provide no support for constraining the short-term use of opioids in the treatment of acute pain.

Two recent studies provide a different picture. The study of Shah et al. (67) involved 1,294,247 patients randomly selected from the IMS Lifeline+ database, which is representative of the US commercially insured population. Among persons prescribed opioids for at least 1 day, the probability of continued opioid use at 1 year was 6.0% and at 3 years, 2.9%. However, because this study involved all patients prescribed opioids and not just those prescribed opioids for a particular medical event, e.g., surgery, it was likely to have included patients with chronic pain whose opioid therapy happened to be initiated during the study interval. Indeed, those maintained on opioids for >1 year were more likely to be older, female, and to have a pain diagnosis before opioid initiation. It also appears that as few as two opioid prescriptions could have defined “continued opioid use” in this study (68).

Brummett et al. (69) reported a retrospective cohort study of 31,177 patients in the Clinformatics Data Mart who underwent major or minor surgical procedures and had not received opioids during the prior year. The primary outcome measure, “new persistent opioid use,” was defined as the filling of one or more opioid prescriptions between 90 and 180 days after surgery by patients who had received a perioperative opioid prescription. Of those undergoing minor surgery, 5.9% met the outcome criterion, whereas of those undergoing major surgery, 6.5% met the criterion. History of back pain, neck pain, arthritis, anxiety, depression, or alcohol or substance use were independently associated with opioid use. Whether or not the filling of as little as one opioid prescription between 90 and 180 days after surgery should be a source of medical concern is unclear. The impact of opioids on pain other than that due to surgery could have informed some patients of their effect on other painful conditions.

Finally, in a systematic review and meta-analysis of 33 studies involving 1,922,743 individuals [which included the Sun et al. (65), Shah et al. (66), and Brummett et al. (69) studies], Lawal et al. (70) found an overall risk of chronic opioid treatment after surgery of 6.7%. However, when the analysis was restricted to opioid-naïve patients, the rate was 1.2%. The major statistical predictors of chronic opioid treatment were pre-operative opioid use, back pain, fibromyalgia, depression, and anxiety.

In summary, the major studies of long-term opioid use after surgery are in substantial agreement that long-term post-surgical rates of opioid use are very low (1% or less), taking into account some variability in the definition of what constitutes extended opioid use and the nature of the surgery. Chronic pain related to pre-existing conditions or to sequelae of surgery are just as plausible as OUD as a potential explanation for long-term opioid use after surgery, although this matter requires further study. One important weakness of the cohort studies we have described is that they cannot tell us how many patients prescribed short-course opioids for medical reasons “went off the grid” and obtained further opioids from illicit sources. This is a difficult population to study and to gain insights requires studies like that of Winkelman et al. (71) (see below: Who are the victims of the opioid crisis?).

## Morbidity and Mortality Associated With Chronic Pain

The intended and unintended effects of the CDC guideline have created a second crisis, this one involving patients in chronic pain (2). Epidemiologic studies suggest that 22% of U.S. adults (55 million) experience chronic pain (3). In any given year, 14.3% of insured adults have pain sufficient to lead to an opioid prescription (3% for >90 days) (72). The corresponding figure for Medicare Advantage patients is 25.7% (7% for >90 days) and for disabled Medicare patients 51.5% (14% for >90 days).

The health-related quality of life of patients with chronic pain is comparable to that of patients dying with cancer (73). Inadequate treatment of chronic pain is associated with increased functional limitations, reduced employment, increased absence from work, disability retirement, reduced household income, poor global recovery from surgery, worsened mental health, increased use of health care resources, increased mortality (3, 74, 75), impaired cognitive function (76), and brain atrophy (77). Chronic pain is associated with increased risk of suicidal ideation, planning, and attempts (78, 79), even after control for psychopathology (80). Chronic post-operative pain impacts activities of daily living in ~25% of patients a year after undergoing inpatient orthopedic surgery (81). Inadequate pain relief after surgery is associated with increased length of stay, re-admission rates, and time to ambulation (64). In 2011, the Institute of Medicine estimated that the annual cost to society of chronic pain, including post-operative pain, was $560–635 billion (82), based on estimated health care expenditures and costs of lost productivity. Treatment of pain has been associated with improvements in activities of daily living, reduced depression or improved mood, reduced fatigue, improved sleep, improved level of function, increased ability to work, increased enjoyment of life, and improved quality of life (3).

These considerations are germane to studies that seek to determine if chronic opioid use is associated with excessive morbidity or mortality. Patients prescribed opioids are likely to differ from those prescribed alternative treatments, pharmacologic or non-pharmacologic, for chronic pain in two ways: (1) they are receiving opioids, and (2) they have more severe pain. If more severe pain is eventually sufficiently mitigated with titrated opioid treatment, then theoretically, mortality attributable to inadequately treated pain should decline with time. Ray and colleagues (83) reported a retrospective study of 45,824 Tennessee Medicaid enrollees, contrasting the mortality associated with treatment with long-acting opioids with that associated with non-opioid analgesics. It provides some support for this hypothesis: the hazard ratio for death during the first 30 days of long-acting opioid prescription was 4.16 but it declined over time to 1.03 in patients on these drugs for >180 days.

Mechanisms of death associated with chronic pain, with or without opioid treatment, have not been adequately studied. They could include cardiovascular events (83) related to stress and heightened sympathetic tone, opioid effects on the heart (83), pulmonary embolism linked to physical inactivity, suicide, and death from overdose of illicit drugs. More generally, comparative cohort studies, even those employing propensity matching [e.g., Solomon et al. (84)] are likely to conflate effects of treatment (e.g., opioids vs. NSAIDS) with effects of disease (more vs. less severe pain) unless the cohorts are adequately matched for pain severity, something impossible to do in retrospective studies.

## Effectiveness of Non-Pharmacologic Treatments

The first of the CDC's 12 recommendations for treatment of chronic non-cancer pain was “Non-pharmacologic therapy and non-opioid pharmacologic therapy are preferred” (1). This statement implies that there is evidence that (1) non-pharmacologic therapies are beneficial for chronic non-cancer pain, and (2) the balance of benefit and risk for these therapies is superior to that achieved with opioids. The benefits and risks of opioid therapy were reviewed above and the risk of harm with non-pharmacologic therapies is likely to be low. Therefore, we will focus on the issue of effectiveness of non-pharmacologic therapies.

In 2018, the Agency for Healthcare Research and Quality (AHRQ) systematically reviewed the literature on non-pharmacologic therapies (85); 4,996 candidate trials were considered and ultimately, 218 publications (representing 202 RCTs) that met quality criteria were analyzed in detail. Most enrolled patients had at least moderate baseline pain intensity (>5 on a 0–10 scale). Most trials reported only short-term outcomes. Treatment was compared with usual care or sham therapy. Treatments reviewed included yoga, tai chi, qigong, spinal manipulation, acupuncture, laser therapy, ultrasound, exercise, massage, multidisciplinary rehabilitation, psychological therapies, cognitive behavioral therapy, mindfulness-based stress reduction, and the Alexander technique for mindful reduction of tension. The strength of the medical evidence, with few exceptions, was graded as low. To the extent that any of these therapies had an effect on pain or level of function, most effect sizes were small. None of the reviewed papers were from Phase III trials. A 2020 AHRQ update did not reveal any important new findings (86). A recent retrospective analysis assessed the impact of massage, acupuncture, and chiropractic care administered over 3 years in a population of 309,277 veterans with chronic musculoskeletal pain, 7,621 of whom received one or more of the therapies (87). There was no significant difference in the self-rated pain intensity outcome between those who were treated and those who were not.

However, we suggest that there is a more fundamental problem with the CDC recommendation: sound scientific grounding for this recommendation would require conducting comparative effectiveness trials. Given the results discussed in the foregoing, it seems unlikely that non-pharmacologic therapies will ever achieve the level of effectiveness needed to justify their use as a sole treatment for moderate to severe chronic non-cancer pain. Rather, their value may lie in their ability to complement pharmacological therapy (including opioid treatment) and thereby reduce drug dosage. The results of 60 small studies (88), almost all targeting short-term treatment of pain, provide some support for this concept. The effectiveness of non-pharmacologic therapies could be tested with a variant of the EERGW design discussed above. Opioid dosage would be gradually reduced to the extent possible in *both* groups. The statistical analysis would compare ultimate opioid dosage in the drug + non-pharmacologic therapy group with that of the drug only group.

## Simultaneous Use of Opioids and Benzodiazepines

The CDC guideline (1) proscribes the concurrent use of opioids and benzodiazepines. Two studies warrant particular attention. Sun et al. (89) conducted a case-cohort analysis of 315,428 patients in the Marketscan database (Truven Health Analytics, Ann Arbor, MI) who filled at least one prescription for an opioid between January 1, 2001 and December 31, 2013. The adjusted odds ratio for an emergency room visit or hospital admission for ostensible opioid overdose among those using both classes of drug was 2.14. Unfortunately, this impressive study suffers a serious methodological weakness: the gold-standard diagnosis derived from physician judgment, not response to naloxone treatment. The alleged risks of concurrent use of these two drug classes have been sounded for many years despite the absence of adequate data. The very fact that a patient is taking the combination may alter the diagnostic evaluation of and the attribution of cause for altered mental status (90). Thus, it is possible that in many patients included in the study by Sun et al., the mere discovery that a patient was taking both an opioid and a benzodiazepine increased the likelihood of a diagnosis of opioid overdose. Consistent with this hypothesis, the diagnosis of opioid overdose related to the combination was made twice as often in 2013 as it was in 2001.

In a case-cohort study, Park et al. (91) analyzed opioid-associated mortality rates in 420,386 veterans prescribed opioids, 27% of whom had concurrent or past prescriptions for benzodiazepines [ssee also Xu et al. (92)]. The past prescription cohort was included in an attempt to control for excess mortality associated with underlying conditions, such as chronic anxiety disorder, post-traumatic stress disorder, and depression, for which benzodiazepines are commonly prescribed and in which drugs are more commonly misused. The adjusted hazard ratio for death in the prior prescription group was 2.33 and in the current prescription group, 3.86. It was elevated for all benzodiazepines except temazepam. Hazard ratio increased with increasing opioid dosage and increasing benzodiazepine dosage. Because of the challenge of controlling for the differences between the benzodiazepine and non-benzodiazepine cohorts, the authors concluded: “benzodiazepines might be better conceptualized as a marker of risk with unknown direct causal links to death from overdose.” Dasgupta et al. (41), in a population-based cohort study of all North Carolina residents, reported a 10 times elevated risk of death associated with the presence of both opioids and benzodiazepines at time of death. However, 49.6% had no active opioid prescription at the time of death, suggesting that in half of the cases, illicit or diverted drugs must have played a role. Therefore, benzodiazepines could have either contributed to risk of death or simply been a marker for polysubstance abuse.

Zedler et al. (93) reported a case-control study (10 controls/case) of 817 VA patients who experienced either opioid overdose or serious opioid-induced respiratory depression. They did identify benzodiazepines as a risk factor (RR 1.49) but also found that antidepressants were actually a greater risk factor (RR 1.98). These findings are consistent with the conclusion of Park et al. (91) that benzodiazepines are best viewed as a marker of risk with unknown direct causal links to the outcome measure.

From these studies, we conclude that calculating the additional risk posed by co-administration of benzodiazepines with opioids poses a major scientific challenge and currently available data can best be considered as suggestive of a modest increase in risk (relative risk ~2), at least part of which may be attributable to concurrent disease rather than the drug combination.

Finally, the CDC guideline did not consider the prevalence and negative impacts of idiopathic insomnia and anxiety disorders or the paucity of effective and safe alternative treatments for these two disorders.

## The Relationship of Depression to Chronic Pain and Its Treatment

Between 30 and 54% of people with chronic pain also have major depressive disorder (MDD) (94). Patients with moderate to severe pain have a more than 2-fold increase in the risk of developing a mood or anxiety disorder (95). General practitioners detect on average 50% of cases of depression (96). Rates of depression reported in large opioid database studies range from 12.9 to 32% (97, 98), suggesting that depression is also commonly missed in patients being treated for chronic pain. Diagnosed depression is often untreated or under-treated (99).

As Braden et al. (100) put it, “It is possible that opioids prescribed to depressed persons may be treating an undifferentiated state of mental and physical pain.” If depression can be viewed as an amplifier of suffering related to pain, then successful treatment of depression might be expected to reduce chronic pain and reduce opioid dosage (a hypothesis testable in a trial employing an EERGW design).

Among patients with chronic pain, inadequately treated depression is associated with a number of adverse outcomes. Patients with depression are three times as likely to be prescribed opioids as those without (100). Among 10,311,961 patients who received short term opioid treatment of pain, depression was associated with a doubling of the hazard ratio for long-term opioid use (101). Patients with depression who are prescribed opioids for non-cancer pain are likely to receive higher doses (100, 102). MDD is associated with a higher prevalence of alcohol use disorders (103) and of opioid misuse and OUD (57, 104). Patients with comorbid non-cancer pain and depression have higher pain interference with activities of daily living and higher mental distress (100). These studies, in aggregate, suggest that aggressive treatment of depression, in addition to its salutary effects on pain management, might mitigate many of the most troublesome issues associated with treatment of chronic pain in patients with comorbid depression.

## The Causes of the Opioid Crisis

**Figure 2:**
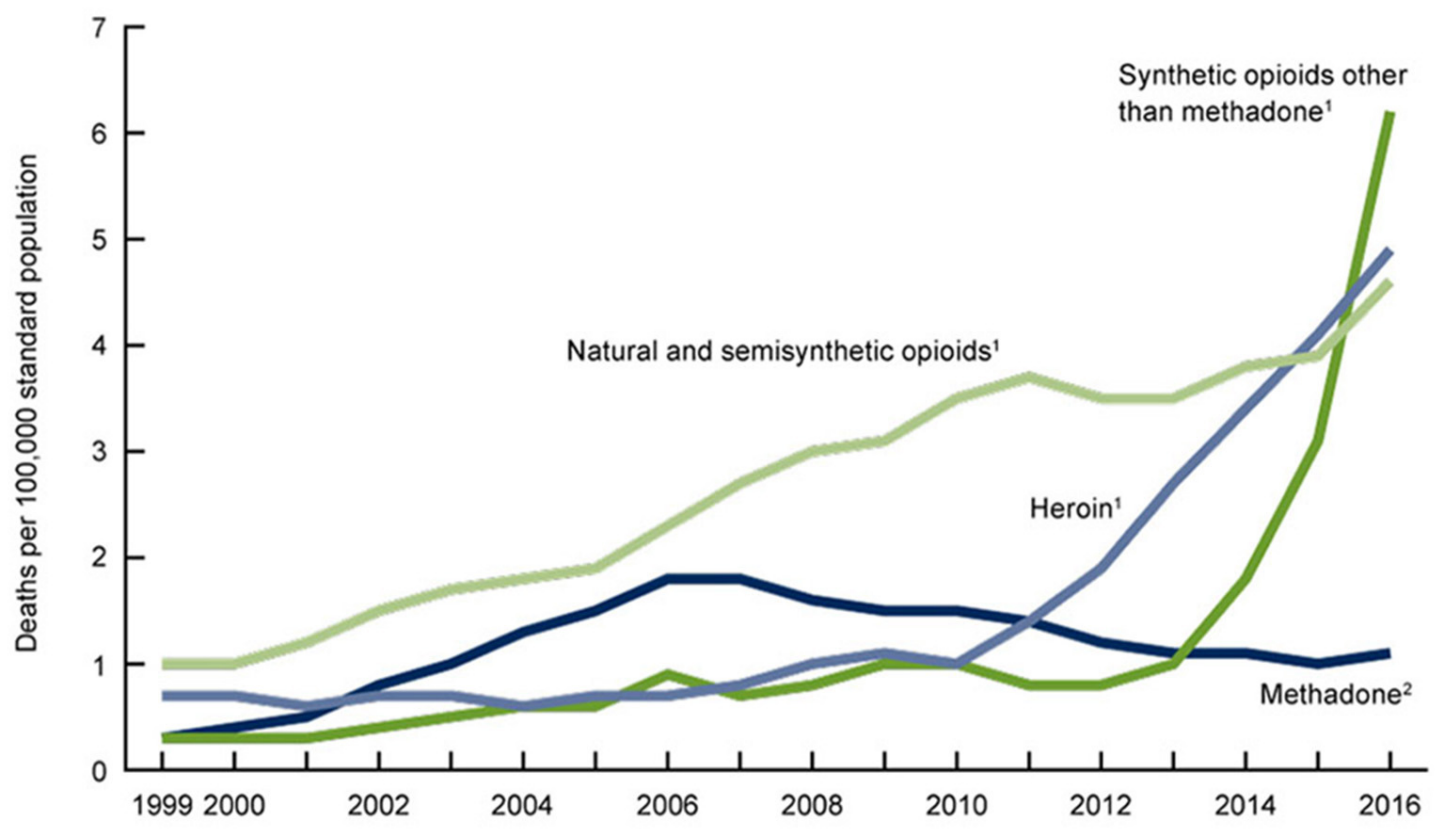
Age-adjusted drug overdose death rates by opioid category, United States 1999–2016 (105). The category of synthetic opioids corresponds almost entirely to fentanyl. A Massachusetts study determined that 96% of this fentanyl was illicitly manufactured (106).

The trends evident in Figures 1, 2 suggest that the opioid crisis has been defined by two separate epochs, the first, which might be termed the “*prescription epoch*,” extending from 1999 to 2011, and the second, which be might termed the “*illicit epoch*,” extending from 2012 to the present. Many efforts to address the opioid crisis, including those by the CDC (1), appear to have conflated the operative mechanisms in these two epochs (107), even as the CDC now explicitly recognizes them (108).

**Figure 3:**
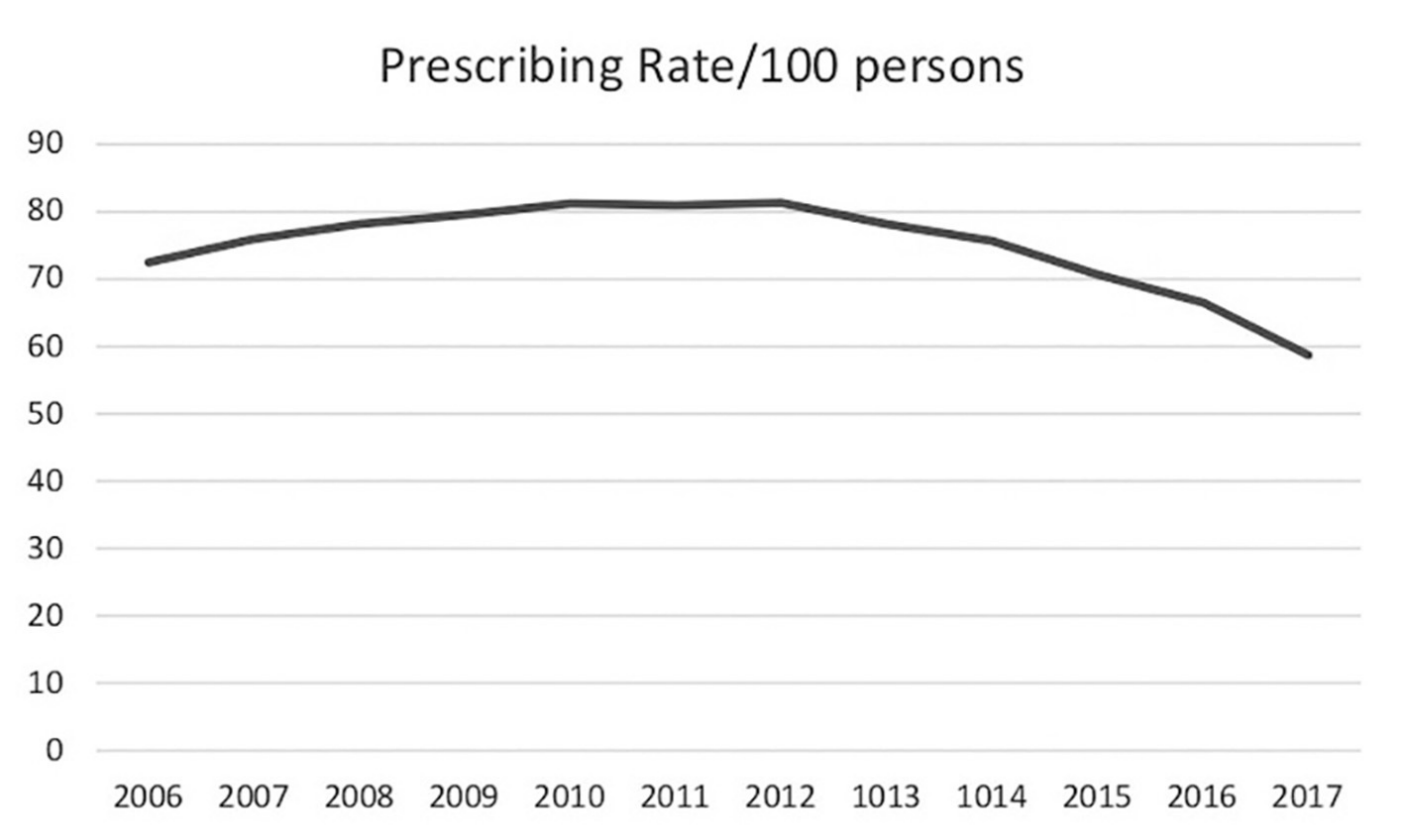
United States annual opioid prescriptions rates/100 persons (110) (CDC data).

Deaths attributed to prescription opioids steadily increased from 6,500 in 1999 to 17,500 in 2011 and have since remained fairly stable. Deaths from illicit opioids roughly doubled between 1999 and 2011, from ~3,000 to 7,000, but were still far outnumbered by deaths associated with prescription opioid use. However, between 2011 and 2015, during the period when mortality from prescription opioids remained stable, deaths from illicit opioids increased from 7,000 to 20,000 and they have continued to rise since (Figure 1). In 2019, there were 49,860 opioid deaths, 13,501 (27%) from prescription opioids and 36,359 (73%) from illicit opioids (109). Opioid prescribing rates peaked in 2012 and have steadily declined since (Figure 3), providing further evidence that prescription practices and deaths from illicit opioids are not linked.

**Figure 4:**
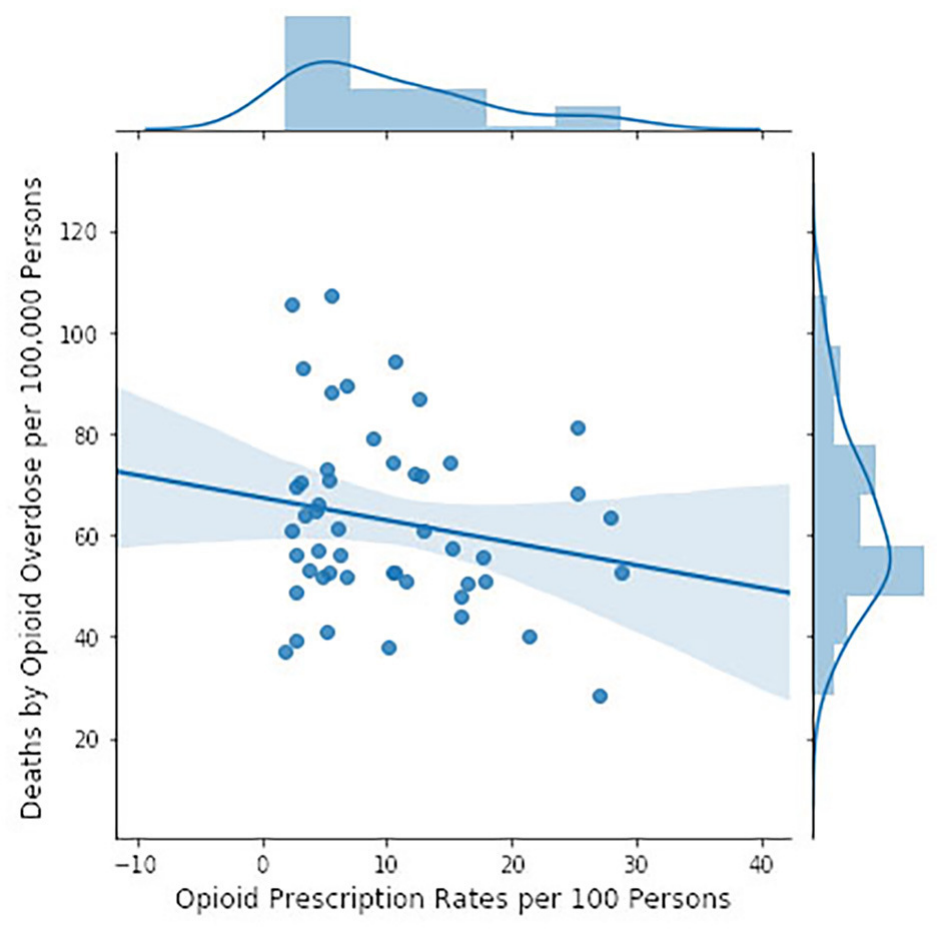
Opioid mortality rates by state in 2016 (111) in relation to number of opioid prescriptions by state (110) (CDC data). Opioid mortality rates are based on ICD codes for narcotic related (T40.0–T40.6) intentional and unintentional drug overdose deaths (X42, X62). Opioid prescriptions accounted for 1.5% of the variance in opioid mortality. The slope of the regression line is not significantly different from zero (*F* = 1.744, *p* = 0.193).

However, the CDC hypothesis is that the opioid crisis has been and still is being driven by excessive prescribing rates. We tested this hypothesis by using state by state data provided by the CDC on prescription rates (110) and mortality (111) (Figure 4). The hypothesis received no support from these data: higher prescription rates were actually associated with lower mortality rates but the adjusted *r*^2^ was only 0.015 and there was not a significant probability that the slope was different from zero.

**Figure 5:**
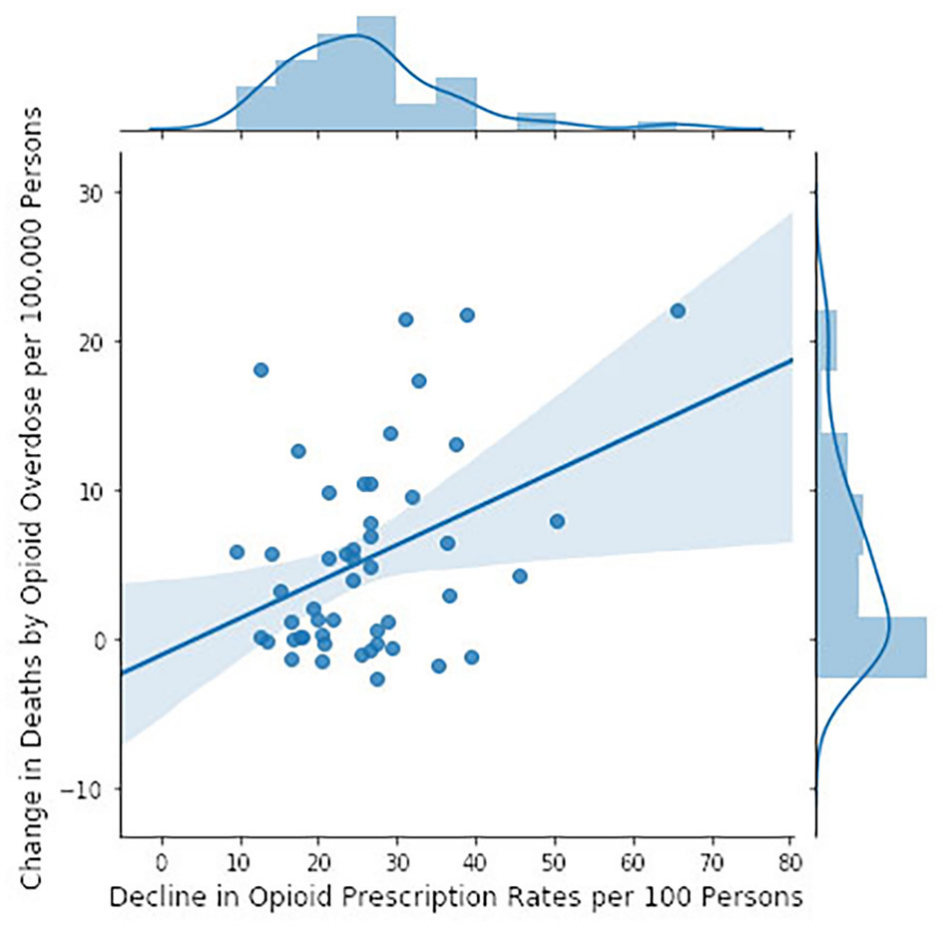
Changes in mortality rates by state (calculated as in Figure 4) between the year of maximal prescription rate and 2017 (111) in relation to the decline in opioid prescription rates from their maximum in the 2006–2016 epoch to 2017 (110) (CDC data). The model accounted for 13% of the variance. The slope of the regression line is significantly different from zero (*F* = 8.298, *p* = 0.00596).

We tested the CDC hypothesis in another way, identifying, state by state, the year of maximal opioid prescribing between 2006 and 2017, which in nearly all states was between 2010 and 2014 (111). We then subtracted the 2017 prescription rate from the rate for that peak year and tested whether this difference correlated with changes in mortality over the same time period (108) (Figure 5). The greater the decline in prescription rates, the greater the *increase* in mortality rates. The adjusted *r*^2^ was 0.132 and there was a significant probability that the slope was non-zero (*p* = 0.00596). Statistical association is not causation, these are multidimensional issues, and there may be other explanations. Nevertheless, if opioid over-prescription has been driving the crisis since 2012, then there should be a correlation between prescription and mortality rates and curtailing prescriptions should be reducing morality; the evidence presented here, derived directly from CDC data, suggests that neither is the case.

If mainstream opioid prescription practices have not propelled the increase in opioid mortality since 2011, then what has? The answer appears to be well-intended efforts by the states to curb pill mills and the ready availability of pure and inexpensive Mexican heroin and Chinese fentanyl, likely complemented by use of opioid pills diverted from other users or obtained from black market sources. Remaining pill mills, aided by the major drug distribution firms that supply them (112), are likely to be an additional factor. What these sources have in common is that they result in use of opioids in the absence of close medical supervision. The evidence is circumstantial but compelling (62, 107, 113–115). Recent data also suggest that an increasing percentage of deaths attributed to prescription opioids likely involves patients who supplemented prescription opioid regimens with heroin and/or fentanyl (115).

There are remarkably little statistical data on pill mills. In fact, one can only infer their prevalence and output from CDC state maps of prescriptions/100 persons (110). Nonetheless, it appears that pill mills were responsible for flooding much of the country with large supplies of prescription opioids, likely starting in the late 1990s (116). It also seems likely that it was the aggressive promotion of Oxycontin (which was FDA licensed in 1996) by Purdue Pharma that made the early phase of the national opioid crisis (2000–2012) more of an Oxycontin crisis than a morphine, hydromorphone or fentanyl crisis.

Because the use of drugs distributed by pill mills was not closely supervised medically, misuse, diversion, and addiction appear to have become prevalent, both in states with large pill mill distributions and in areas of the country that were most susceptible to the lure of opioids because of poverty, mental illness, hopelessness, and a complex of other factors (117). Deaths from pill mill prescribed drugs also likely made a substantial, albeit incalculable, contribution to the rising mortality from prescription opioids between the late 1990's and 2012. Unfortunately, because state efforts to reign in pill mills came late, by 2012, the population of people who were misusing or were addicted to prescription opioids had grown to substantial proportions. In a 2007 study of 27,816 individuals entering addiction treatment programs, 78% of those who reported use of Oxycontin also reported that the drug had not been prescribed for any medical reason (118). As pill mill crackdowns became more prevalent, these people either could not obtain prescription opioids or could no longer afford them. The introduction of an abuse-deterrent formulation of Oxycontin in 2010 may also have been a factor (62). In states with the highest initial rates of Oxycontin misuse, the introduction of the abuse-resistant formulation was associated with the largest differential increases in heroin deaths (119).

The pill mill crackdown and the introduction of abuse-resistant Oxycontin brought the prescription epoch to an end. The introduction of inexpensive and easily available high purity heroin and fentanyl, often in combination, appears to have then ushered in the illicit epoch, playing a major role in increasing unsupervised opioid use and associated mortality (58, 62, 120). Heroin use has always been dangerous but, because of the 50 times greater potency of fentanyl and the unpredictable amount of lacing of heroin with fentanyl, heroin use has been converted from merely dangerous to something akin to Russian roulette, hence the continued and accelerating climb of opioid deaths since 2011.

In summary, one would not have expected a strategy to control the opioid crisis to be effective when it consisted of restriction of physician prescribing practices in a crisis actually caused by pill mills, black market opioids, and street heroin and fentanyl. The evidence suggests that indeed, this has been failed approach.

## Who Are the Victims of the Opioid Crisis?

Because the people caught up in the crisis live substantially off the medical grid until they die, it is difficult to know exactly who they are. However, a recent study (71) has provided a great deal of information about this population. Winkelman et al. analyzed data from the 2015–2016 NSDUH sample (see above, Prevalence of Opioid Use Disorder). Of the sample, 23,452 (29.7%) interviewees had used prescription opioids, 3,913 (4.95%) reported prescription opioid misuse, 648 (0.82%) prescription abuse (defined by DSM-4 criteria), and 451 (0.57%) heroin use. The misuse/abuse/heroin use population was preponderantly male (respective percentages in the three groups 54.2, 58.9, and 66.9); white (65.8, 72.9, and 72.3%); aged 34 or less (particularly the heroin users); high school or less educated; single (63.9, 68.4, and 85.7%); without children; had income below twice the federal poverty level; and listed themselves as unemployed or other. These individuals were more likely to report fair or poor health (15.5, 24.9, and 17.6%); a chronic health condition; any disability (22.5, 35.7, and 26.0%); and impairment of mental health (particularly in abuse and heroin use groups). They were 3–4 times as likely as non-opioid users to report alcohol dependence or abuse (19.2, 25.6, 16.2%). They were far more likely to use other drugs, including sedatives, tranquilizers, stimulants, hallucinogens, inhalants, methamphetamine, cocaine, or marijuana (68, 82.7, and 92.9% reported use of one or more).

These use data fit quite well with data on annual age-specific opioid-related overdose mortality, bearing in mind that overdose-related mortality is complex, multiple drugs are commonly implicated in any given death, assay of some drugs is complicated and may be beyond the capabilities of individual medical examiners, and death may not be accurately attributable to a single drug, or even be due to drugs. Between 2012 and 2017, mortality rose from 8 to 18/100,000/year in the 15–24 age group; 7.7–16.8 in the 25–34 age group; 9–15 in the 35–44 age group; 5.3–10.8 in the 45–54 age group; 1.2–2.2 in the 55–64 age group; 0.6–0.8 in the 65–74 age group; and 0.5–0.7 in the 75–84 age group (111). Thus, the age groups most likely to be *prescribed* opioids for chronic pain, seniors over age 55 (121), and that likely benefited the most from the liberalized opioid prescription policies of the 1990 s and early 2000's, experienced low mortality rates and a small absolute increase in opioid-overdose related mortality, whereas mortality was high and rose rapidly among younger people who are infrequently prescribed opioids for longer than a few days.

A complex array of factors contributes to opioid abuse (113), including poverty, lack of opportunity, substandard living and working conditions (and the contribution of job-related injury to pain and downward mobility), unstable housing, imprisonment for drug-related offenses, childhood adverse experiences, poor physical and mental health, social isolation, and the development of hopelessness and despair—all quite congruent with the data of Winkelman et al. (71). The age patterns of opioid use cited above suggest that young people are particularly likely to respond to these factors with opioid abuse.

## Impact of CDC 2016 Guideline on Health Care Providers

The CDC guideline issued in 2016 (1) was ostensibly intended to guide the opioid prescribing practices of primary care physicians. The mechanisms underlying their actual effect appear to have escaped serious scrutiny as we have been unable to find any systematic studies [however, see (107)]. Nevertheless, it is our impression that the guideline has achieved its greatest impact by convincing health care provider organizations that violations of the guideline by their member physicians may increase organizational liability exposure (114). Because suspension of clinical privileges—a catastrophic outcome for individual physicians—can be easily accomplished, limitation of prescription dosage, and even participation in comprehensive pain management is under near absolute control by these organizations. In addition, by mid-2017, 23 states had passed laws limiting prescription duration or dose or authorizing other entities to set limits with effective legal force (122). In all but four of these states, these laws were limited to prescriptions for acute pain. However, we suggest that this intrusion of state legislatures into pain management may have further reduced the willingness of physicians to provide comprehensive pain management. Of note, in June 2020, The American Medical Association (123) publicly suggested that the CDC guideline could be substantially improved by the urging of state legislatures, payers, pharmacy chains, pharmacy benefit management companies, and all other stakeholders to immediately suspend use of the CDC guideline as an arbitrary policy to limit, discontinue or taper a patient's opioid therapy.

## Conclusions

This analysis of the clinical scientific literature on opioids suggests that many of the conventional assumptions about opioids, including safe opioid dosage, opioid efficacy, the factors that lead to opioid use and abuse, and the risks associated with opioid use, are not supported and in many cases, are refuted by existing scientific data. Conclusions about opioid efficacy, or the lack thereof, have been drawn from seriously flawed RCTs characterized by inadequate experimental designs. Data on the high variability in opioid dosage requirements and the high frequency of idiosyncratic side effects have been overlooked. Estimates of the risk of death from prescription opioids have been largely predicated on the national increase in total opioid mortality from all sources, legal and illegal. Well-designed studies have demonstrated estimated annual case fatality rates for >100 MMED regimens in the vicinity of 0.25%/year—a level of risk comparable to that associated with chronic anticoagulation for prophylaxis of stroke due to atrial fibrillation. Excess risk of death associated with opioid use conflates risks attributable to opioids and risks related to being in chronic pain, with its associated comorbidities. Risks of the development of OUD have commonly been overestimated, even as the operational definition of OUD requires further research. State legislatures are passing laws based on the gateway theory even as scientific evidence has demonstrated that this theory has little merit.

Strong measures are being taken to restrict prescription opioid use without consideration of the vast cost of inadequately treated chronic pain, whether measured in terms of human suffering and degraded quality of life or in terms of the literal costs of health care and lost productivity ($600 billion/year). Ideas about the potential value of alternative non-pharmacologic therapies have flourished despite the lack of comparative effectiveness studies. Absolute proscription of co-prescription of opioids and benzodiazepines appears to have effectively become the law of the land, even as studies supporting this concept have yielded data that are at best suggestive. These studies have also revealed the complexity of this issue. The relative effectiveness and risks of alternatives to benzodiazepines for treatment of idiopathic insomnia and anxiety have received no consideration. The potential role of depression in contributing to the adverse effects of chronic pain and its treatment and the potential value of aggressive treatment of depression in chronic pain patients have scarcely been considered.

The causes of the opioid crisis are now coming to light and a coherent narrative can be constructed. It seems that the CDC, in attempting to deal with a crisis in the streets by restricting treatment of pain in clinics, has created a second very serious crisis, this one involving 18 million patients in moderate to severe chronic pain. These CDC efforts have not addressed the crisis in the streets, one now accounting for nearly 3/4 of opioid deaths (109). This is a crisis of community economic failure, poverty, social isolation, hopelessness, and serious mental health problems.

Clearly it is time to return to the scientific evidence bearing on these issues, of which there is a considerable body. We now have a fairly clear picture of what needs further study. Innovative RCT designs have been proposed, e.g., EERGW, to test opioid efficacy and dosage variability, to conduct comparative effectiveness studies, and to assess the impact of comorbidities such as depression. Much is known about how to treat opioid addiction. What is lacking is adequate funding and implementation of treatment programs. Management of chronic pain is complex, labor intensive, requires considerable investment of health care resources, and entails significant risk. Major improvements in training of physicians (45), health care infrastructure, and re-imbursement policies are needed to optimize care and minimize risk.

## Author Contributions

SN researched and wrote the manuscript. Much of the manuscript reflects an ongoing scientific dialogue between SN and RL over the course of 3 years. RL reviewed and critiqued the manuscript. JW was responsible for the statistical analyses. All authors have reviewed the manuscript and concur in its content.

## Conflict of Interest

The authors declare that the research was conducted in the absence of any commercial or financial relationships that could be construed as a potential conflict of interest.

## Publisher's Note

All claims expressed in this article are solely those of the authors and do not necessarily represent those of their affiliated organizations, or those of the publisher, the editors and the reviewers. Any product that may be evaluated in this article, or claim that may be made by its manufacturer, is not guaranteed or endorsed by the publisher.
